# A Low-Cost IoT System for Real-Time Monitoring of Climatic Variables and Photovoltaic Generation for Smart Grid Application

**DOI:** 10.3390/s21093293

**Published:** 2021-05-10

**Authors:** Gustavo Costa Gomes de Melo, Igor Cavalcante Torres, Ícaro Bezzera Queiroz de Araújo, Davi Bibiano Brito, Erick de Andrade Barboza

**Affiliations:** 1 Computing Institute, A. C. Simões Campus, Federal University of Alagoas—UFAL, Maceió, AL 57072-970, Brazil; icaro@ic.ufal.br (Í.B.Q.d.A.); davi@ic.ufal.br (D.B.B.); erick@ic.ufal.br (E.d.A.B.); 2 Center of Agrarian Sciences, Engineering and Agricultural Sciences Campus, Federal University of Alagoas—UFAL, Rio Largo, AL 57100-000, Brazil; igor.torres@ceca.ufal.br

**Keywords:** monitoring, data acquisition systems, renewable energy

## Abstract

Monitoring and data acquisition are essential to recognize the renewable resources available on-site, evaluate electrical conversion efficiency, detect failures, and optimize electrical production. Commercial monitoring systems for the photovoltaic system are generally expensive and closed for modifications. This work proposes a low-cost real-time internet of things system for micro and mini photovoltaic generation systems that can monitor continuous voltage, continuous current, alternating power, and seven meteorological variables. The proposed system measures all relevant meteorological variables and directly acquires photovoltaic generation data from the plant (not from the inverter). The system is implemented using open software, connects to the internet without cables, stores data locally and in the cloud, and uses the network time protocol to synchronize the devices’ clocks. To the best of our knowledge, no work reported in the literature presents these features altogether. Furthermore, experiments carried out with the proposed system showed good effectiveness and reliability. This system enables fog and cloud computing in a photovoltaic system, creating a time series measurements data set, enabling the future use of machine learning to create smart photovoltaic systems.

## 1. Introduction

The share of renewable energies in electricity generation has been growing worldwide. In 2019, there was an increase of 200 gigawatts of renewable energy in the world energy matrix, with photovoltaic energy being responsible for 57.5% of this increase according to [1]. Small and medium-sized distributed photovoltaic generation systems were the ones that grew the most. In Brazil, at the end of 2020, distributed generation represented 59% of installed photovoltaic sources, with a 107% growth compared to 2019, while centralized generation had an increase of only 24% [2].

The expansion of distributed renewable energies presents several benefits, such as less environmental impact, reduced emission of carbon dioxide, and less degradation of fauna and flora. Regarding social impacts, this type of generation system can be employed in remote locations that do not have access to the power grid, enabling and improving access to communication, education, and agricultural production. Renewable energies, especially solar energy, tend to generate more jobs than non-renewable energy generation, and less centralized systems can create more opportunities.

A primary feature of photovoltaic (PV) systems is the correlation between the climatic conditions and the performance of its generation. The availability of sunlight, temperature and various other climatic factors directly affect energy production. In large and medium-sized centralized photovoltaic systems, many of the efforts and resources are used in monitoring and acquiring data, which is essential to recognize the renewable resources available on-site, evaluate the efficiency of electrical conversion, detect failures and optimize electrical production.

On the other hand, in small photovoltaic systems, the high monitoring cost generally makes its implementation inaccessible. The high cost can lead to situations in which the system operators do not detect failures such as loss of efficiency, peaks and falls in voltage, and insertion of harmonics in the power grid [3,4]. These failures can disrupt the operation of the photovoltaic system or even cause damage to the power grid [5].

The introduction of the Internet of Things (IoT) concept to monitoring devices can bring several benefits, such as access to real-time data, remote device management, cost reduction, and system scalability. Moreover, it allows the integration of devices into the smart grid, enabling improvements in the photovoltaic system’s processing, fault recovery, and reliability skills [6]. Furthermore, wireless communication decreases the distance range limitation and costs typical of wired communication.

This paper presents the design, development, and validation of a low-cost IoT data acquisition system that focuses on real-time monitoring of photovoltaic energy generation and the main meteorological factors that influence the generation. The principal motivation is to provide an alternative solution for commercial systems that are usually expensive and closed to adjustments and modifications. The proposal consists of three main elements: (1) two data logger devices for data acquisition, one for meteorological data and other for PV generation data; (2) an IoT cloud system that processes and stores the data obtained; (3) and a web application that displays the real-time data and the previous data collected.

The proposal includes improvements in software, hardware, and system architecture (IoT). To the best of our knowledge, considering the similar works found in the literature, this is the first proposed PV monitoring system that aggregates all of the following features:

- Measurements of all the relevant meteorological variables;
- Open software implementation;
- LoRa (Long Range) as the data transmission technology and connection with the internet without cables;
- Data storage locally and in the cloud;
- Network time protocol (NTP) to synchronize the devices’ clocks;
- PV generation variables measured directly from the plant, not from the inverter.

The focus of hardware development was flexibility and cost reduction. Furthermore, with the application of the IoT cloud system, the proposed system allows remote control, local and cloud storage of data, real-time access to data, and scalability. Due to these features, the system is more oriented to small/medium operators than distribution system operators (DSOs). Furthermore, it can be of interest to researchers because it provides an enabling technological system at an affordable price. Moreover, it can be of high interest to professionals working in developing countries where the limited diffusion of solar technology can be attributed to lack of funding and research and development activities [7].

It is worth mentioning that this is an enabling system for creating intelligent photovoltaic systems. It provides an IoT architecture that enables machine learning techniques to be executed using cloud or fog computing paradigms. Moreover, the data sets generated by the system can be used to train machine learning algorithms for fault detection and power generation forecasting, for example.

The rest of the paper is structured as follows. Section 2 describes the proposed system with details about the device’s hardware, the operation of the devices, the LoRa protocol developed, the IoT architecture used, and the web application developed. Section 3 presents a review of related works in the literature. Section 4 presents the results and discussions with a technical comparison of the proposed system with previous systems, experimental results for the validation of the proposed system, and cost analysis of the proposed system. Finally, Section 5 provides the conclusions.

## 2. Proposed System Description

### 2.1. System Overview

**Figure 1:**
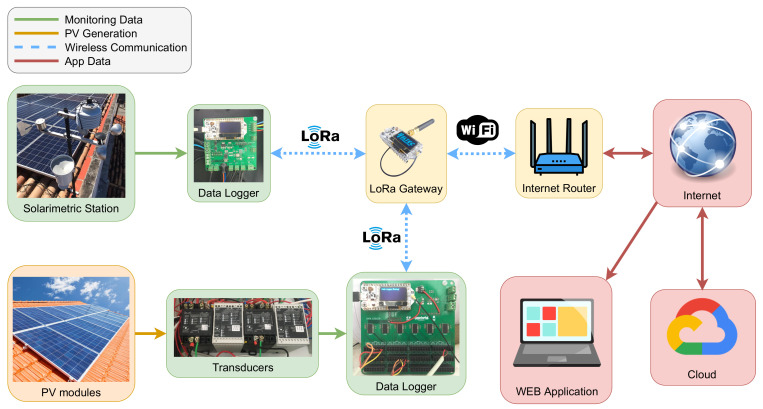
Simplified diagram of the proposed system.

Figure 1 shows an overview of the system. The data logger devices are responsible for collecting, conditioning, storing, and transmitting data from all sensors. We developed two different devices. The first one is responsible for collecting the meteorological data from the solarimetric station sensors. The second one is a data logger to monitor the PV generation.

The data logger devices use LoRa wireless communication to send the data obtained to a LoRa gateway, and Wi-Fi (IEEE 802.11) connects the gateway to the internet. The gateway is the intermediary between the devices and the cloud system. It is responsible for redirecting the monitored data to the cloud, storing it, or directing commands and settings from the cloud to the devices.

All data acquired by the system can be accessed easily through the web application, allowing real-time viewing of data or querying data stored in the cloud database. A remote server hosts the web application, enabling users to access it from any browser.

### 2.2. Data Logger Devices

When designing the data logger devices, the goal was to achieve low production costs, provide wireless communication, and be flexible for software and hardware changes. The main component of these devices is the Heltec Wi-Fi LoRa 32 (V2) IoT dev-board [8], which features the ESP32 dual-core microcontroller (MCU) [9] and integrates Wi-Fi, LoRa, Bluetooth (IEEE 802.15.1), onboard OLED display, and micro-USB connector.

The solarimetric station data logger requires a robust MCU to operate in harsh environments since the station will be exposed to different climatic conditions. The ESP32 was built for use in industrial environments. It can work in temperatures between −40 and 125 °C and adapt dynamically to external condition changes.

Each data logger has an SD card (secure digital card), where the obtained data are saved temporarily before sending it to the gateway. This local storage ensures that data are not lost if communication with the gateway or the cloud is not available. The 74HC125D buffer [10] performs communication between the MCU and the SD through a serial peripheral interface (SPI) bus.

Furthermore, a real-time clock (RTC) was used to monitor the date and time of each measurement, providing an accurate time when the MCU will read the sensors’ data. The RTC communicates directly with the MCU through an inter-integrated circuit (I2C) bus and has a dedicated battery allowing the time to be tracked continuously even if the data logger is without power.

#### 2.2.1. Solarimetric Station Data Logger and the Meteorological Variables

**Figure 2:**
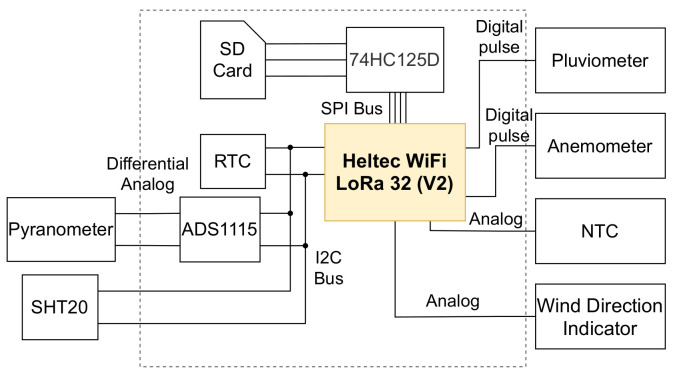
Simplified diagram of the solarimetric station data logger, with emphasis on the components and connections.

Figure 2 shows the solarimetric station data logger’s main components and the connections between them. This device is responsible for monitoring the following variables: irradiance, PV module temperature, wind speed and direction, ambient temperature, humidity, and rain.

Solar irradiance is one of the essential meteorological variables. The energy generated by a photovoltaic system is directly proportional to the irradiance that reaches the photovoltaic modules. A low-cost pyranometer measures solar irradiance. The relationship between pyranometer input and output is given by Equation (1). *G* is the irradiance incident, *K* is the calibration constant of the pyranometer, and mV is the output voltage in millivolts. Since the pyranometer generates a very low voltage, the high precision ADS1115 analog-to-digital converter (ADC) [11] was used to read the measurements.

(1) $$G=\frac{mV}{K}$$

The photovoltaic module temperature influences its photovoltaic conversion efficiency. It was estimated at [12] that 0.5% PV module efficiency is reduced with an increase of 1 °C in its temperature. The data logger uses a 10 kΩ negative temperature coefficient (NTC) sensor to measures the PV module temperature. The relationship between the resistance of the NTC (*R*) and its temperature ($T_{m}$) is provided by Equation (2), where $R_{25}$ is the resistance of the NTC at the reference temperature, $T_{25}$ is the reference temperature (25 °C), and β is the NTC constant.

(2) $$T_{m}\left(R\right)=\frac{1}{\frac{ln\left(\begin{array}{c} \frac{R}{R_{25}} \end{array}\right)}{\beta }+\frac{1}{T_{25}}}$$

The ambient temperature influences the PV module temperature, and air humidity can absorb or reflect solar energy, decreasing the irradiance that reaches the photovoltaic module. The SHT20 sensor [13] measures these two factors. Rain can affect many factors at the same time, such as reducing solar irradiance and panel temperature. The data logger obtains the rainfall index through a rain gauge.

The wind can help reduce the PV module temperature, improving its efficiency. An anemometer measures wind speed, which generates a digital pulse at each turn around itself. The accumulated pulses (CP) during a given period (*P*) are used to calculate the revolutions per minute (RPM), as shown in Equation (3). The revolutions per minute are then converted to km/h using Equation (4), where *r* is the radius of the anemometer. The wind direction measurement is performed by a wind direction indicator with an analog output that varies according to the direction the indicator is pointing.

(3) $$RPM=\frac{CP\times 60\times 1000}{P}$$

(4) $$W_{s}=\frac{4\times \pi \times r\times RPM}{60\times 1000}\times 3.6$$

#### 2.2.2. PV Generation Data Logger

**Figure 3:**
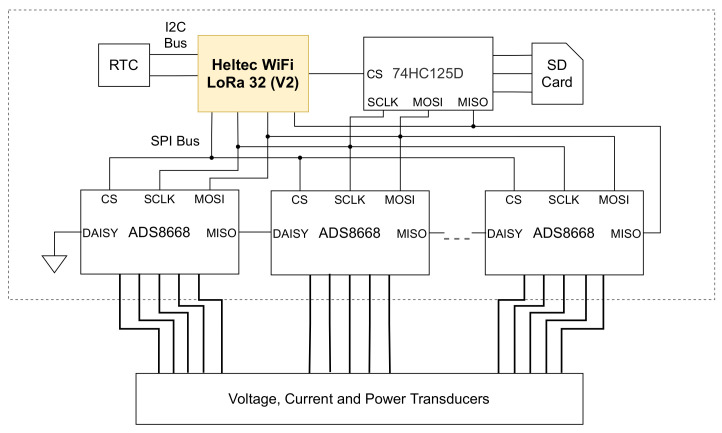
Simplified diagram of the PV generation data logger, with emphasis on the components and connections.

The PV generation data logger is responsible for monitoring the direct current (DC) voltage and current of multiple PV strings and active alternating current (AC) power at the inverter output. To achieve this, six ADC ADS8668 [14] were used. Each ADS8668 has eight channels of differential analog input and communicates with the MCU through the SPI bus. To establish the communication of the six ADCs with the MCU using only one chip selector pin, a unique topology called daisy-chain was used, as illustrated in Figure 3.

During the experiment and validation of the system, five SECON transducers [15] were used, two for current, two for voltage, and one for power. The current transducer proportionally converts an input current between 0 and 10 A into a voltage between 0 and 5 V. The two voltage transducers have different measurement ranges; the first reads between 0 and 400 V and the second between 0 and 500 V. Both have a proportional output between 0 and 10 V.

(5) $$P=V_{out}\times 3600-9000$$

A ±9 kW bidirectional three-phase transducer measures the AC power. This transducer can receive power up to 9000 W as input and provides an output voltage between 0 and 5 V. Because the transducer is bidirectional, the relationship between its output and measured power is provided by Equation (5), where *P* is power, and $V_{out}$ is the transducer output voltage.

### 2.3. Data Loggers Operation

**Figure 4:**
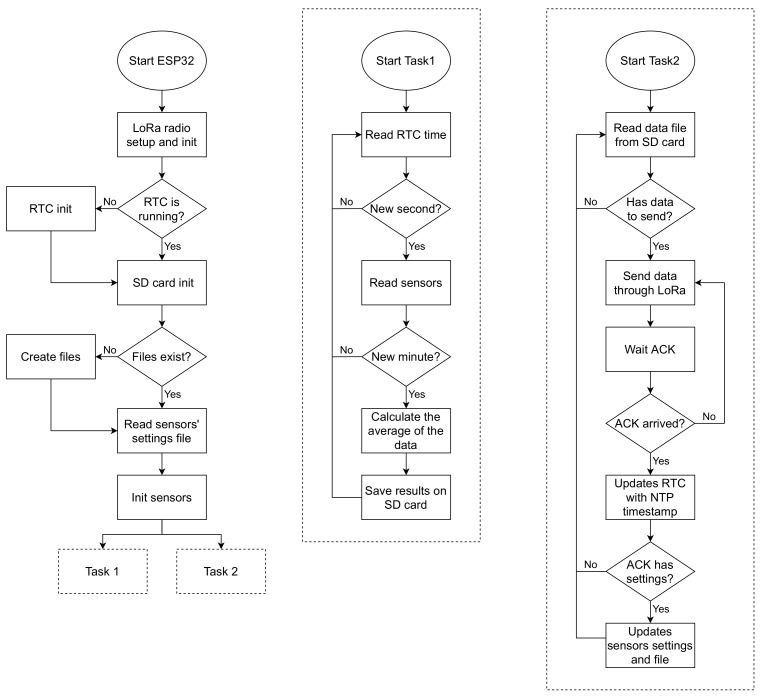
Simplified diagram representing the operation of the data logger devices.

Figure 4 shows a flowchart illustrating the main steps in the operation of the devices; both data loggers operate similarly. When powered on, the data logger first initializes the LoRa radio, RTC, and SD card. Then, the existence of the data and settings files on the SD card is verified, and if these files do not exist, they are created in a comma-separated values (CSV) format. The data file is created with a header that informs the data type in each file column. The configuration file is created with the default sensor settings.

Next, all connected sensors are initialized, and the saved settings are applied to some of those sensors (transducers and the pyranometer). After the initialization of the sensors, two tasks are assigned to each of the ESP32 cores to run in parallel.

The first task is responsible for reading the sensors’ data. It contains a loop that performs polling of the time marked by the RTC. If the time obtained second value is different from the last sampling second value, a new sampling is performed, and each of the obtained data is added to the value stored in its respective variable. When the second equals 00, the values accumulated in the variables are divided by the number of samples, averaging the last-minute data (60 samples). This approach is based on [16].

In short, the data are sampled every second for one minute, and then an average is calculated. This method is not applied to the monitoring of rain, which is the daily accumulation, and the direction of the wind, determined by the most frequent direction in that last minute. After calculating the average, a timestamp is obtained from the RTC. Then, the results and timestamps are saved in the SD card data file, and the storage variables are reset to zero.

The second task is performed every five seconds (using the function delay) and manages LoRa communication. This interval was defined based on tests to obtain a high send frequency without interfering with the execution of the first task. Each time the second task is executed, it is checked if there are data on the SD card to be sent. If data are detected on the SD card, the first data set in the file is compressed, added to a LoRa packet, and sent to the LoRa gateway. After, the task expects to receive an acknowledgment (ACK) that the data have reached the gateway. If confirmation does not arrive within a specific time, the data are resent. When the ACK is received, the data set sent is removed from the SD card, and a new set is prepared for sending.

Therefore, in regular operation, data are sent from the data loggers to the cloud every minute. In the event of a communication failure, upon returning, the accumulated data will be sent every five seconds until all data are sent and the operation is normalized.

The LoRa gateway is always synchronized with the NTP.br server [17]. When an ACK is sent to a device, a timestamp obtained through the NTP is sent with it. The NTP timestamp is used by the second task to update the RTC date and time. Thus, the RTC will always maintain the correct time with great precision. This RTC update also allows the devices’ time to be synchronized, causing data sampling to occur roughly at the same time.

Sensors’ configurations may also be included in the ACK package. These settings can be set in the cloud system and contain constants used in the initialization and the reading of the transducers and the pyranometer, allowing remote adjustments of the measurements. The second task applies the settings and saves them to the SD card file to be maintained even if the data logger is restarted or loses communication with the cloud.

### 2.4. LoRa Network Protocol

A custom LoRa network protocol was developed specifically for the proposed system to send the data in the most efficient way possible. The Arduino-LoRa library [18] was used for both devices and at the gateway to transmit and receive LoRa packets. This library exposes the LoRa radio directly and allows it to send data to any radios in range with the same radio parameters, without using compression or addressing.

Two functions offered by the library were used: sync word and cyclic redundancy check (CRC). The sync word function limits data transmission only to devices that share the same sync word value, creating an isolated LoRa network. CRC is an error detection method to detect an accidental change in the transmitted data packets.

**Figure 5:**
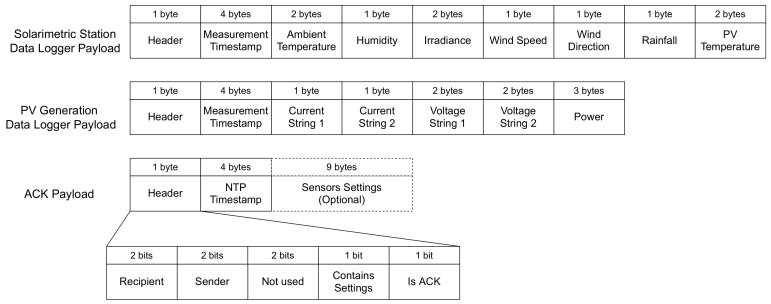
Proposed LoRA payload structure.

A payload structure was developed to maximize data transfer using the smallest number of bits. Figure 5 shows the structure for transmitting sensor data and ACK information. The first byte is a header that contains the information of whom the sender and recipient are, performing the addressing, and also contains bits that inform if the payload is an ACK and if it contains sensor settings information.

The data are compressed using a bit-packing, so its representation uses fewer bits than if they were transmitted as ASCII code. Bit-packing is a simple compression, where the data are first represented in an integer value using the bit packing formula, and then it is represented in binary. Table 1 details this representation for each type of data.

As Arduino-LoRa only supports transmitting char (1 byte in C language), even the pieces of data less than 8 bits were allocated one byte in the structure. This allocation also facilitates the separation of the data in the receiver.

### 2.5. IoT Architecture

**Figure 6:**
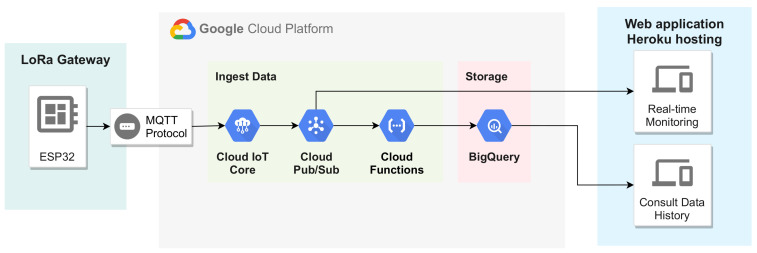
Diagram representing the IoT architecture.

The data collected by the proposed system are made available to the user through a cloud system. For this project, the Google Cloud Platform (GCP) [19] was used to implement the IoT architecture. However, Amazon Web Service, Microsoft Azure, or any other cloud system could also be used. Figure 6 illustrates the proposed IoT architecture.

The GCP IoT Core module is responsible for managing the devices and defining which communication protocol they can use. IoT Core provides the options to register, update, and monitor the devices’ status as a device manager. In this proposal, the LoRa gateway was registered as an IoT device, and a key pair was generated to perform the device authentication and secure communication. The generated public key was registered in the IoT Core while the private key was implemented in the LoRa gateway.

For communication between the GCP and LoRA gateway, the protocol “message queuing telemetry transport” (MQTT) is used. MQTT is a machine-to-machine communication protocol based on the publish/subscribe pattern to exchange asynchronous messages. The Pub/Sub module is the GCP MQTT broker, responsible for managing topics and subscriptions. Pub/Sub offers temporary message storage and real-time message delivery with high availability and consistent performance on a large scale.

Two MQTT topics were created, one to receive data sent from the station and the other to receive PV generation data. Each of these topics has two subscribers. The first subscriber is the real-time monitoring page of the web application, allowing a real-time data display to the user. The second subscriber is the cloud functions module of GCP, which transfers the data to BigQuery for storage.

The GCP BigQuery module is a standard query language (SQL) database for large data sets. A table was created to store the data for each data logger device. The web application can make queries to BigQuery based on dates and obtain a data history for display.

The IoT Core automatically creates two topics for each registered device, a configuration topic and a command topic. The configuration topic is used to transmit the sensor settings of the data logger devices, and the command topic can be used to reset the LoRa gateway remotely. Both topics are accessible only through the IoT Core web page.

### 2.6. Web Application

The web application provides a simple and easy way for the user to view and obtain the data acquired by the proposed system. The application was developed using the Django Web framework, which uses Python to manage and render web pages. In Python, four endpoints were created to access the cloud services using the GCP SDK: two endpoints for subscription in the MQTT topics of each data logger and two others to query the BigQuerry tables.

**Figure 7:**
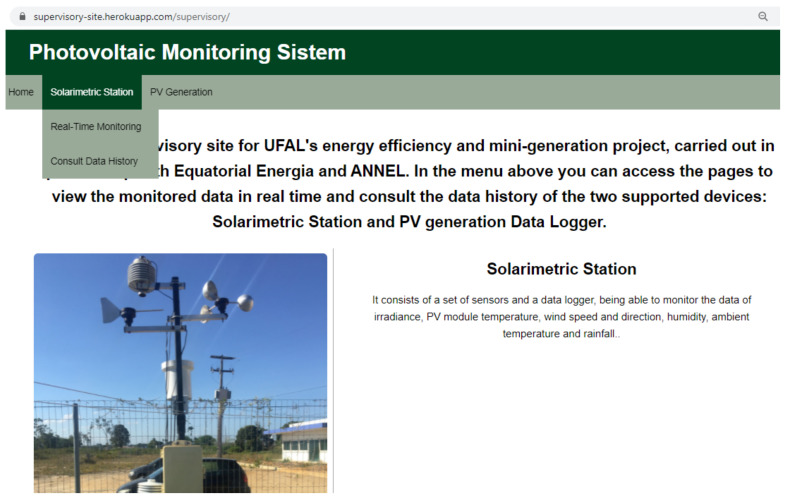
Web application home page, displaying the drop-down sub-menu.

Three web pages were implemented using HTML and JavaScript: home page, real-time monitoring, and consult data history. Figure 7 shows the applications home page that contains some information about the work developed. Each device has a real-time monitoring page and a consult data history page, accessed from the top menu. When the cursor passes through the devices’ names in the top menu, a drop-down sub-menu is displayed to select the desired page.

**Figure 8:**
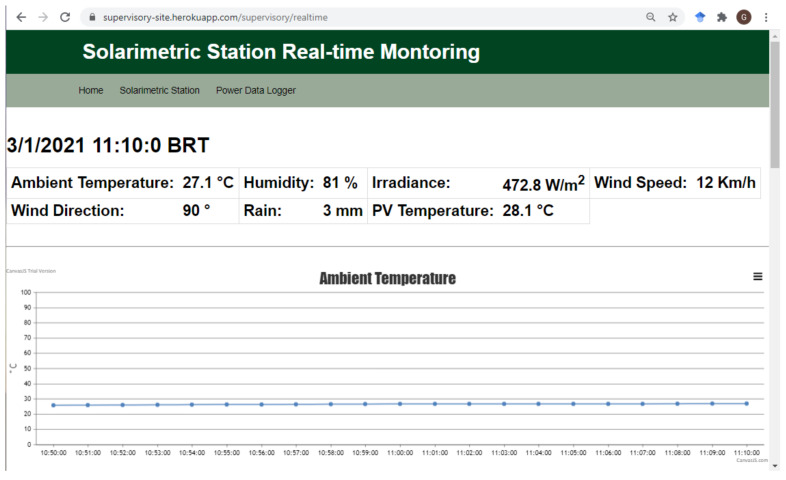
Web application page for real-time monitoring of the solarimetric station.

The real-time monitoring page uses one of the Python endpoints to subscribe to the device’s data topic. As soon as the data are received through the MQTT, they are displayed in two sections of this page. The first section shows the latest data set that arrived, including the date and time it was obtained. In the second section, each monitored variable is displayed in a different line chart. The charts begin to display the data received from the moment the page is opened and can display up to 1440 points simultaneously (24 h of monitoring). When the maximum point limit is reached, the oldest points are removed as new data are received. Figure 8 shows the real-time monitoring page of the solarimetric station, with the first section and the first chart of the second section.

**Figure 9:**
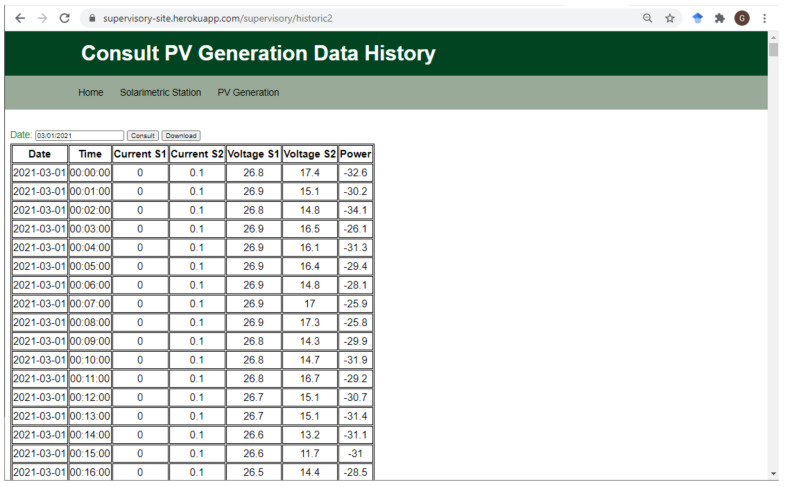
Web application page to consult the history of PV generation data.

On the consult data history page (Figure 9), the user can choose a day, manually typing in MM/DD/YYYY format or using an interactive calendar to consult the data saved in BigQuery. When choosing the date and pressing the “Consult” button, the Python endpoint queries the table in BigQuery, and the data obtained, if any, are displayed on the page in table format. The “Download” button converts the data displayed in the table into a CSV file transferred to the user’s computer.

The web application is hosted on Heroku [20], a cloud platform as a service with support for different profile languages, including the Django framework.

## 3. Literature Review and Comparison of Monitoring Systems Applied to PV Plants

Several related works can be found in the literature. Despite the advantages and advances of the literature systems presented below, they all have at least one limitation. Table 2 presents a comparison of some of the technical characteristics of the systems available in the literature and the system proposed in this work. In the rest of this section, we will discuss the similarities and differences between the proposed system and the related ones.

The monitoring system proposed in [21] consists of two types of devices: smart meter and main brain. Smart meters are the devices responsible for monitoring the voltage and current data of the PV system in real-time, while the main brain is the center where the data collected by the devices smart meter will be stored. Both are based on the ATmega 328P-PU MCU and communicate via a radio frequency (RF) wireless network operating at 315 MHz. The data can be accessed by a mobile application that communicates with the main brain via Bluetooth.

In [22], a two-level sensor network was developed for monitoring PV systems. The first level of the network consists of sensor nodes that monitor the voltage and temperature of each PV module. In contrast, the second level consists of sensor nodes that monitor irradiance, ambient temperature, voltage, and current of each string. In addition, the second-level nodes merge their monitored data with the data obtained by the first level and send it to a data center. The communication between the levels is via a radio frequency wireless network, and the second level uses ZigBee [23] to send all the collected data to the center.

Internet connection is crucial to provide real-time monitoring of data and to allow remote access to the system. In [21,22], the data are only available locally. Local-only availability would also make it difficult for future integration of these systems into a smart grid network. Internet-connected monitoring systems can be configured in two different topologies: the data logger devices connect directly to the internet or intermediate devices between the internet and the data loggers.

The authors of [24] developed a wireless sensor network based on the ESP8266 MCU to monitor the photovoltaic system. Each network node monitors the current and voltage data of a set of photovoltaic modules and connects to the internet via Wi-Fi to send the collected data to an IoT cloud platform. The system is also capable of monitoring the humidity and temperature of the solar plant.

In [25], the proposed system is based on ESP32 and ESP8266, which communicate with an unspecified cloud system via Wi-Fi. Data for temperature, irradiance, humidity, wind speed, and DC generation are collected every 47 s and are made available through a web application.

Initially, the proposal’s data loggers were configured to communicate with the internet via Wi-Fi, similarly to [24,25]. However, this approach presented a limitation in the positioning of data logger devices due to the Wi-Fi range. Thus, the second topology using LoRa and Wi-Fi was adopted in the current version.

Aghenta and Iqbal [26] present an IoT approach that focuses on monitoring PV generation without performing meteorological data acquisition. An Arduino performs sensor data acquisition and communicates with a Raspberry Py via a serial bus. The Raspberry Pi is connected to the internet via an Ethernet cable through which the collected data are sent to an IoT platform based on a local server, where it is stored and can be accessed.

The system proposed in [27] monitors voltage, current, temperature, and irradiance. This system is based on an Arduino and uses a Raspberry Py as a gateway. The two devices communicate using the I2C protocol, while the Raspberry Py communicates with a cloud service using the MQTT protocol. In addition to storing and making data available, the cloud service can also send configuration commands to devices.

Regarding data transmission between devices, in [26,27], short-distance wired communication was used, limiting the disposition of devices and making installation more complex.

The system presented in [28] has a structure based on wireless sensor networks, in which each sensor node monitors the current and voltage generated by an individual photovoltaic module. The nodes send data via ZigBee to a Raspberry Py that hosts a web page, allowing access to data locally and over the internet via Wi-Fi.

In [29], ZigBee modules are used to collect and transmit data obtained from the PV plant inverters, building a local sensor network. A 4G gateway is used to connect the local network to the internet, enabling remote data access. Checksum verification is used to ensure the stability of the data transmission and to verify its integrity.

ZigBee technology generally has a range of 10 to 100 m and low energy consumption. The LoRa typically has a range of 2–5 km in urban areas or 15 km in suburban areas and has an even lower energy consumption than ZigBee. These were the main reasons for the adoption of LoRa in the proposal. However, LoRa has a lower data transfer rate than ZigBee.

LoRa is also used in the system implemented in [30] for data transmission. The system can monitor DC and AC electrical data, the temperature of the PV modules, irradiance, ambient temperature, and humidity. A LoRa gateway is responsible for making the data available on a local network to be accessed from a computer.

A few studies have reported a method for synchronizing the clocks of the devices that make up the system. The clock synchronization is essential for systems that use a single device and systems composed of multiple devices, allowing the measurements to have a correct timestamp and accurately represent the events in the PV plant.

A system for fault detection in PV systems is presented in [31]. The National Instruments CompactRIO (cRIO) controller is used to obtain the solar irradiance and ambient temperature data from a weather station and the DC and AC voltage and current data from the PV system. The collected data are then used in techniques for detecting and classifying faults in the PV system. The cRIO clock is updated through the LabVIEW software.

In [32], precision time protocol (PTP) (IEEE 1588) was used to synchronize the timestamps of the slides that make up a wireless sensor network. The network comprises wireless sensors that monitor irradiance, ambient temperature, the temperature of the PV modules, rainfall index, wind speed and direction, atmospheric pressure, DC and AC electrical data.

Network time protocol was used in the proposed system due to its easy access to information and because it is widely used in applications that require a precise timestamp. In [25], the NTP was also used, as the devices connect directly to the internet, it is only necessary to access the date and time information using the IP address of the NTP server. In the proposal, for the NTP data to be transmitted to the data logger devices, it was necessary to integrate it into the LoRa payload.

The system proposed in [33] is based on PcDuino (discontinued), which combines Arduino with Raspberry Py operating on Linux, being able to monitor temperature, irradiance, wind speed and direction, and AC and DC electrical data. The data are stored locally on an SD card and accessed over the internet. Storing data only locally on the system can create difficulties and a greater complexity when providing remote access. This form of storage is performed in [21,22,26,28,31,32].

Storing data only on remote servers can cause data loss if there is a communication failure. This is done in [24,29,30]. Performing both types of storage can prevent these problems and make the system more reliable, as was done in [25,27] and in our proposal.

Commercial software that requires a license is used in [22,31,32]; in addition to making changes to the system challenging, it also makes it more expensive. In the proposed system, the device software is developed in C++ using the Arduino framework, which is open-source and widely used and supported by the community.

Finally, for complete monitoring of a PV system, it is necessary to monitor: (1) the meteorological factors to which the system is subjected; (2) the DC electrical generation of the PV modules; (3) and the AC output of the inverter. This is accomplished in [30,31,32,33]. Our proposal also involves acquiring these three types of data. Furthermore, dedicated sensors are used without relying on data provided by the inverter. Dedicated sensors allow the proposed system to be applied to any PV system and independent of the sampling of data provided by the inverters. The systems in [25,29,33] are dependent on the inverter.

## 4. Results and Discussions

### 4.1. Experiment

The proposed system was applied to monitor a PV microsystem consisting of 19 polycrystalline silicon (Si-p) modules. Each module has a nominal power of 270 Wp (watt-peak) and can be associated in series with a nominal 5130 Wp. The PV system was configured in two strings due to the maximum voltage limitations of the inverter used. String 1 has ten panels associated in series, and String 2 has nine panels also associated in series. The connection of the strings is made through a DC/AC inverter with a nominal power of 5000 W, reaching a maximum peak of 6500 W.

**Figure 10:**
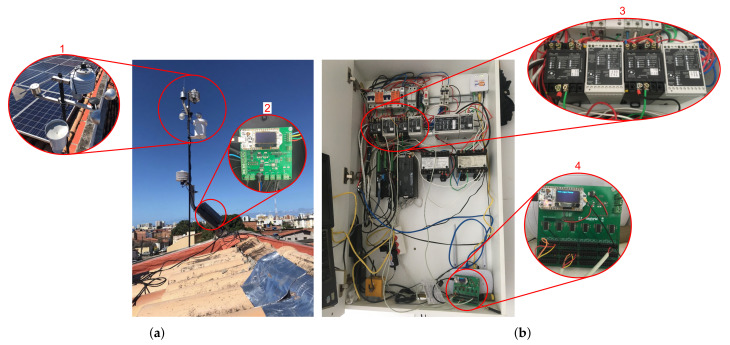
Proposed system installed in a PV plant. (**a**) Solarimetric station, with emphasis on its data logger and sensors. (**b**) Cabinet with the transducers and the PV generation data logger.

Figure 10 shows the system installed on-site. Figure 10a shows the solarimetric station discussed in Section 2.2.1, with highlight 1 showing the pyranometer, anemometer, rain gauge, and wind direction indicator. The SHT20 is positioned inside a weather shelter in the middle of the station’s structure. The NTC is fixed to the back of one of the PV modules. The solarimetric station data logger is contained in an airtight box for protection and is shown in highlight 2.

The cabinet shown in Figure 10b is located next to the inverter and has devices for protection and sectioning of the PV plant. Highlight 3 shows the DC voltage transducers (white) and DC current (black). Next to them is the AC power transducer. As detailed in Section 2.2.2, the data from the transducers are acquired by the PV generation data logger (highlight 4). The following results refer to the data monitored from 27 February 2021, to 14 March 2021.

### 4.2. Proposed System Operation

The architecture of the proposed system has good reliability and was effective in displaying collected data in real-time. During the 16-day experimental period, 23,040 data sets from each data logger were expected to be collected and sent. A total of 99.13% of the data sets from the solarimetric station, and 99.40% of the PV generation data sets, reached the cloud, demonstrating the system’s reliability.

All data that arrived at GCP were successfully saved in BigQuery and sent to the web application via MQTT. Regarding the effectiveness of being in real-time, calculating the average of the data obtained by task 1 until these data are displayed in the web application is fast, with a delay of at most five seconds. Most of this delay is introduced by executing task 2 of the data loggers every five seconds, which is necessary to maintain the consistency of the device’s operation.

The use of dedicated sensors allows the proposed system to be applied to other photovoltaic systems, regardless of the inverter used. Furthermore, the sampling of the proposal does not depend on the sampling of PV generation data provided by the inverter. Sampling every second for one minute, followed by the average of the data obtained in that interval, provides accurate measurements, keeping the transmission, storage, and computation of data in low complexity [16].

The proposed LoRa protocol reduces the size of the payload allowing a more efficient transmission, which reduces the transmission time and energy consumption. Table 3 shows a comparison with the load sizes transmitted by the proposed structure, by LoRaWAN using Cayenne low power payload (LPP) [34] and as a text string.

The use of NTP to synchronize RTCs brought significant advantages. First, it allowed RTCs to have the correct date and time. Before using NTP, the RTC had a small precision error that could accumulate, generating an error of several minutes. Second, it allowed a simple way to synchronize the measurements between the two data loggers.

### 4.3. Measured Data Validation

A comparison with the data collected by a second monitoring system was performed to validate the data collected by the proposed system. This second monitoring system is based on the CR1000 data logger from Campbell Scientific [35] and has external sensors for measuring the ambient temperature, solar irradiance, and temperature of a PV module. The PV generation data are obtained by the same transducers used by the proposed system.

Before performing the comparisons and statistical calculations, a preprocessing of the data was performed. Regarding the data obtained by the proposed system, some of the PV generation values had errors in their measurements (e.g., being outside the expected range). The last valid reading replaced these values. Some current data obtained by CR1000 showed the not-a-number (NAN) value. These values were also removed and replaced by the last valid read value.

After preprocessing, the following statistical metrics were calculated between the data obtained from the two systems: mean absolute error (MAE) (Equation (6)), root mean square deviation (RMSD) (Equation (7)) and weighted absolute percent error (WAPE) (Equation (8)).

(6) $$MAE=\frac{1}{n}\sum_{n}^{i=1}\left|y_{i}-x_{i}\right|$$

(7) $$RMSD={\left[\begin{array}{c} \frac{1}{n}\sum_{n}^{i=1}{\left(y_{i}-x_{i}\right)}^{2} \end{array}\right]}^{0.5}$$

(8) $$WAPE=\frac{\sum_{n}^{i=1}\left|y_{i}-x_{i}\right|}{\sum_{n}^{i=1}\left|y_{i}\right|}$$

where *n* is the number of data samples, $y_{i}$ is the i-th sample of data collected by CR1000, and $x_{i}$ is the i-th sample of data collected by the proposed system. Table 4 shows the results.

MAE measures the average magnitude of the errors between the two data sets without considering their direction. Similarly, RMSD expresses average error, but as the errors are squared before they are averaged, the RMSD gives a relatively high weight to large errors. MAE and RMSD provide the error in units of the variable of interest, which can generate a misleading comparison between the errors of the different measurements. For this reason, WAPE was also calculated, showing the errors as a percentage.

The factors that presented WAPE below 10% were considered acceptable, including ambient temperature (4.13%), currents of the two strings (5.37% and 7.29%), and the AC power (6.6%). Considering the units and magnitudes of these factors, they also presented a low MAE that should not impact the measurement quality. The RMSD of the power (210.9 W) and currents were between 2.4 and 3.2 times higher than the MAE. This relationship between errors may indicate the presence of outliers in these measurements. The irradiance (13.54%), the temperature of the PV module (17.56%), and the voltage measurements (15.15% and 11.99%) showed WAPE greater than the acceptable value. Although, these values can be improved with some adjustments discussed below.

**Figure 11:**
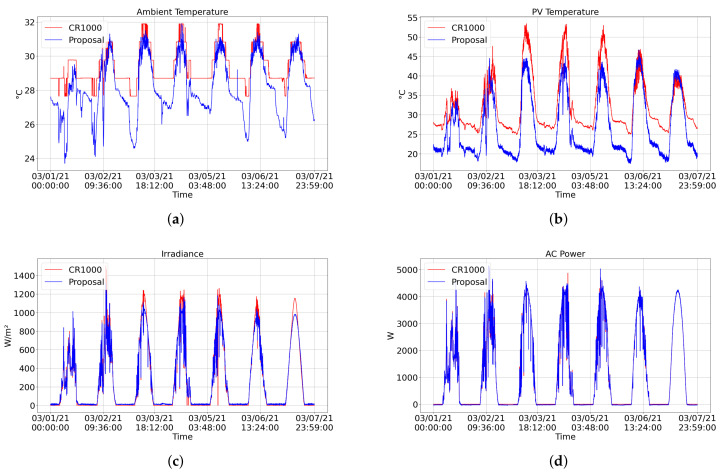
Graphical comparison of the data obtained during one week (1 March to 7 March 2021) by our proposed system (blue) and the CR1000 (red). The graphs show the following measurements: (**a**) ambient temperature, (**b**) PV module temperature, (**c**) irradiance, (**d**) AC power.

**Figure 12:**
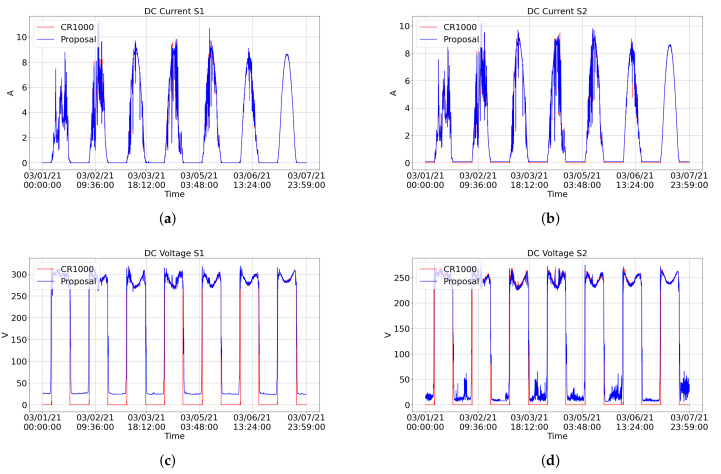
Graphical comparison of the data obtained during one week (1 March to 7 March 2021) by our proposed system (blue) and the CR1000 (red). The graphs show the following measurements: (**a**) string 1 current, (**b**) string 2 current, (**c**) string 1 voltage, (**d**) string 2 voltage.

Figure 11 and Figure 12 shows graphical comparisons of measurements over seven days (1 March to 7 March 2021). The blue line represents the measurements returned by our proposed system, and the red line represents the measurements returned by the CR1000. The CR1000-based system does not monitor data on humidity, wind speed, wind direction, and rain, so they have not been compared.

One can see that the error in the measurement of irradiance is mainly present when this factor reaches its maximum value (Figure 11c). This error can be caused due to the difference in the pyranometers’ installation location. The pyranometer of the proposed system is installed on top of the structure of the solarimetric station (Figure 10a). In contrast, the pyranometer of the CR1000 system is installed at a lower level, next to the PV modules. Furthermore, the irradiance signal of the proposed system also presents noise. The application of a filter can reduce this noise and the error.

Most of the error in voltage measurements is generated during the night, where the proposed station measured values above 0 V when it should be zero (Figure 12c,d). Zeroing the reading below a threshold value would reduce these errors in the voltage readings. The temperature of the PV module showed the most significant error among all factors when using an NTC to monitor these data. In the equation used to convert the resistance presented by the NTC to temperature, expected constants were applied for an ideal 10 kΩ NTC. Performing a calibration to find the specific constants of the NTC used would reduce the error presented.

### 4.4. Cost Description and Comparison

The cost of producing the solarimetric station data logger was USD 65.42, and this value includes the printed circuit board and electronic components, such as resistors, capacitors, voltage regulator, ADS1115, RTC, SD card, and Heltec Wi-Fi LoRa 32 (V2). The production of the PV generation data logger was USD 109.11. The higher price is due to the ADCs that the device contains. The LoRa gateway is a Heltec Wi-Fi LoRa 32 (V2), costing USD 20.80.

The cost of the sensors used are: pyranometer—USD 279.45, NTC 10K—USD 3.14, SHT20 (with waterproof protection)—USD 27.97, anemometer—USD 37.10, wind direction indicator—USD 37.10, pluviometer—USD 48.44, voltage transducer—USD 55.93, current transducer—USD 82.96 and power transducer—USD 236.24. Adding the costs of the data loggers and their sensors, we have a total cost of USD 498.62 for the solarimetric station and USD 623.13 for the PV generation monitors, so the total hardware cost of the proposed system is USD 1142.55, including the gateway.

The production cost can be lower when considering only the components used in the experiment and if the purchase of the components is optimized. For example, only one of the ADCs of the PV generation data logger was used during the experiments. Thus, the remaining ADCs can be removed, reducing USD 44.4, for a total cost of USD 1098.15. Furthermore, as the system is flexible, any sensors or transducers can be easily replaced with cheaper alternatives.

A comparison can be made with the work developed in [33] since it is one of the most complete of the literature and provides the cost of its development. The authors reported a cost of USD 25,000.00 to develop 20 units of the system. Therefore, each unit has a value of around USD 1250.00. To monitor meteorological and PV generation factors, two units of this system are required. Thus, our system is two times cheaper than this one. Another comparison can be made with the CR1000 data logger, the cost of which is about USD 1354.32 (average of eBays offers). The CR1000 does not include any sensors or transducers. Adding these devices to the CR1000, forming a PV monitoring system that monitors the proposed system’s same factors, would be more expensive. Hypothetically, applying the sensors and the transducers used in the proposed system, which cost USD 926.42, to the CR1000 would result in a system with a total cost of approximately USD 2280.74 (CR1000 + sensors).

Regarding the proposed system software, all the code used in the developed devices was open source, adding no extra cost. In relation to GCP, the monthly cost is USD 0.20, based on the amount of data obtained during the month of March 2021 (31 days) and without considering the free monthly use of some of the services. We intend to implement in the future the same IoT infrastructure based on the open-source messaging agent Mosquitto [36], offering a free alternative to GCP. Heroku offers 1000 h per month to run free applications at no cost, so the web application does not add costs to the system.

## 5. Conclusions

In this work, an IoT system was developed for the real-time monitoring of photovoltaic systems. The IoT system comprises two data logger devices, a cloud system, and a web application. It can monitor weather and PV generation data.

The proposed system differential is that it measures all the relevant meteorological variables, is implemented using open software, uses LoRa as the data transmission technology, connects with the internet without cables, storages data locally and in the cloud, uses network time protocol to synchronize the devices’ clocks, and measures PV generation variables directly from the plant (not from the inverter). To the best of our knowledge, no work reported in the literature presents these features altogether.

Moreover, experimental results showed the correct effectiveness of real-time data display and good reliability of the proposed system. The cost of production proved to be low, being almost twice as cheap as a system based on a commercial data logger and one of the complete systems found in the literature. Therefore, the proposed system can be an excellent alternative to micro and mini PV systems. Nevertheless, since it is an open system, it is scalable and easily modified, enabling it to be used in PV systems of different topologies and sizes.

Some of the future works are:

- Implement filters and perform sensor calibration on the proposed system to improve the accuracy of PV module temperature, DC voltages, and irradiance measurements;
- Implement a Mosquitto message broker in a dedicated server to avoid the need for a paid cloud service, which will decrease the data cost;
- We intend to integrate the proposed system with machine learning techniques to forecast photovoltaic generation based on meteorological data and automatically detect failures, allowing optimization of the electrical production process and increase the reliability of the PV plant.

## Figures and Tables

**Table 1:** Data bit-packing.

Data Type	Bit-Packing Formula	Bits Required	Range	Precision
Timestamp	B = seconds from 1 Jan 1970 00:00:00	32	1 Jan 1970 00:00:00 to Jan 19 2038 03:14:07	1 s
Temperature	B = (T + 40) × 10	16	−40 to 125 °C	0.1 °C
Humidity	B = H	7	0 to 100%	1%
Irradiance	B = I × 10	16	0 to 6553.5 W/m^2^	0.1 W/m^2^
Wind Speed	B = S	8	0 to 255 Km/h	1 Km/h
Wind Direction	Maps to a value that represents the direction	3	0, 45, 90, 135, 180, 225, 270, 315°	-
Rainfall	B = R/0.25	8	0 to 63.75 mm	0.25 mm
Voltage	B = V × 10	16	0 to 6553.5 V	0.1 V
Current	B = C × 10	8	0 to 25.5 A	0.1 A
Power	B = (P + 9000) × 10	24	−9000 to 9000 W	0.1 W

**Table 2:** Technical characteristics of cited data acquisition systems. UN means unspecified and refers to features that are present in the system, but the technology used has not been specified. NM means not mentioned and indicates features that were not mentioned in the articles. The abbreviations of the measured parameters are DC current (Idc), DC voltage (Vdc), DC power (Pdc), AC current (Iac), AC voltage (Vac), AC power (Pac), ambient temperature (Ta), PV module temperature (Tm), irradiance (G), humidity (h), pressure (p), rainfall (rf), wind speed (Ws) and wind direction (Wd).

System	Measured Parameters	Open SW	Data Transmission	Internet Connection	Data Storage	Devices Sync	Dedicated Sensors
Caruso et al. [21]	Idc,Vdc	Yes	315 MHz RF	No Connection	Local SD card	NM	Yes
Su et al. [22]	Ta,Tm,G,h,Idc,Vdc	No	RF and ZigBee	No Connection	Local computer	NM	Yes
Al-Naima and Hamad [24]	Ta,h,Idc,Vdc	Yes	-	Wi-Fi	ThingSpeak cloud database	NM	Yes
Pereira et al. [25]	Ta,Tm,G,h,Ws,Idc,Vdc,Pdc	Yes	-	Wi-Fi	Local flash memory and cloud database	NTP	No
Aghenta and Iqbal [26]	Idc,Vdc,Vb	Yes	Serial	Ethernet	EmonCMS local server	NM	Yes
Zedak et al. [27]	Ta,G,Idc,Vdc	UN	I2C	UN	Local Raspberry Pi and cloud database	NM	Yes
Zago and Fruett [28]	Idc,Vdc	Yes	ZigBee	Wi-Fi	Local Raspberry Pi	NM	Yes
Xia et al. [29]	Idc,Vdc	UN	ZigBee	4G	Cloud server database	NM	No
Paredes-Parra at al. [30]	Ta,Tm,G,Idc,Vdc,Iac,Vac	Yes	LoRa	Ethernet	The Things Network cloud server	NM	Yes
Lazzaretti et al. [31]	Ta,Tm,G,h,Ws,Wd,Idc,Vdc,Iac,Vac	No	-	Ethernet	Local database	LabVIEW	Yes
Moreno-Garcia et al. [32]	Ta,Tm,G,p,rf,Ws,Wd,Idc,Vdc,Iac,Vac	No	UN	Ethernet	Local database	PTP	Yes
Erraissi et al. [33]	Ta,Tm,G,Ws,Wd,Idc,Vdc,Pdc,Iac,Vac,Pac	Yes	Bluetooth	Ethernet	Local SD card	NM	No
Proposed system	Ta,Tm,G,h,rf,Ws,Wd,Idc,Vdc,Pac	Yes	LoRa	Wi-Fi	Local SD card and cloud database	NTP	Yes

**Table 3:** Payload size in bytes considering different protocols.

Protocol	Station Data	PV Generation Data	ACK (min)
Proposed	15 B	14 B	5 B
Cayenne LPP [34]	33 B	28 B	9 B
Text String	61 B	56 B	27 B

**Table 4:** Statistical comparison between the measures of the proposed system and the CR1000 data logger considering the 16 days of the experiment and three types of metrics: MAE, RMSD and WAPE.

Data type	MAE	RMSD	WAPE
Ambient Temp.	1.21 °C	1.46 °C	4.13%
Irradiance	37.05 W/m^2^	68.12 W/m^2^	13.54%
PV Module Temp.	5.67 °C	6.73 °C	17.56%
DC Current String 1	0.12 A	0.41 A	5.37%
DC Current String 2	0.17 A	0.41 A	7.29%
DC Voltage String 1	21.46 V	48.02 V	15.15%
DC Voltage String 2	14.51 V	38.23 V	11.99%
AC Power	74.01 W	210.90 W	6.60%

## Data Availability

The data collected by the proposed system are openly available. The data for the solarimetric station can be found in Meteorological Data Proposed System at 10.6084/m9.figshare.14216969. Photovoltaic generation data are available in PV Generation Data Proposed System at 10.6084/m9.figshare.13953845. The data from the CR1000-based system were provided by Igor Cavalcante Torres and can be found in CR1000 Data at 10.6084/m9.figshare.14225111. The software codes of the two data loggers and the of LoRa gateway, as well as the complete schematics of the data logger devices, can be found in the Photovoltaic Monitoring System repository at https://github.com/gustavo95/Photovoltaic-Monitoring-System. All the developed code is authorial, except for the code used to access the Google cloud. Some modifications made to the Google code are documented at the beginning of each modified file, as requested by the Apache 2.0 license. Moreover, the hardware project, design, and assembly were done by the authors.

## Abbreviations

The following abbreviations are used in this manuscript:

PV	Photovoltaic
IoT	Internet of Things
LoRa	Long Range
NTP	Network Time Protocol
DSO	Distribution System Operator
MCU	Microcontroller Unit
RF	Radio Frequency
cRIO	Compact RIO
DC	Direct Current
AC	Alternating Current
PTP	Precision Time Protocol
I2C	Inter-Integrated Circuit
MQTT	Message Queuing Telemetry Transport
SD	Secure Digital
SPI	Serial Peripheral Interface
RTC	Real-Time Clock
ADC	Analog-to-Digital Converter
ACK	Acknowledgment
CRC	Cyclic Redundancy Check
GCP	Google Cloud Platform
SQL	Standard Query Language
CSV	Coma-Separated Values
UN	Unspecified
NM	Not Mentioned
Idc	DC Current
Vdc	DC Voltage
Pdc	DC Power
Iac	AC Current
Vac	AC Voltage
Pac	AC Power
Ta	Ambient Temperature
Tm	PV Module Temperature
G	Irradiance
h	Humidity
rf	Rainfall
p	Pressure
Ws	Wind Speed
Wd	Wind Direction
USD	United States Dollar
LPP	Low Power Payload
NAN	Not A Number
MAE	Mean Absolute Error
RMSD	Root Mean Square Deviation
WAPE	Weighted Absolute Percent Error
